# Mental Health of Pregnant and Postpartum Women During the Coronavirus Disease 2019 Pandemic: A Systematic Review and Meta-Analysis

**DOI:** 10.3389/fpsyg.2020.617001

**Published:** 2020-11-25

**Authors:** Haohao Yan, Yudan Ding, Wenbin Guo

**Affiliations:** National Clinical Research Center for Mental Disorders, and Department of Psychiatry, The Second Xiangya Hospital of Central South University, Changsha, China

**Keywords:** coronavirus disease 2019, pregnant women, anxiety, depression, insomnia

## Abstract

**Background:** Prenatal and postnatal mental disorders can exert severe adverse influences on mothers, fetuses, and children. However, the effect of the coronavirus disease 2019 (COVID-19) pandemic on the mental health of pregnant and postpartum women remains unclear.

**Methods:** Relevant studies that were published from January 1, 2019 to September 19, 2020 were identified through the systematic search of the PubMed, EMBASE, and Web of Science databases. Quality assessment of included studies, random-effects meta-analysis, sensitivity analysis, and planned subgroup analysis were performed.

**Results:** A total of 23 studies conducted with 20,569 participants during the COVID-19 pandemic and with 3,677 pregnant women before the COVID-19 pandemic were included. The prevalence rates of anxiety, depression, psychological distress, and insomnia among pregnant women during the COVID-19 pandemic were 37% (95% confidence interval [CI] 25–49%), 31% (95% CI 20–42%), 70% (95% CI 60–79%), and 49% (95% CI 46–52%), respectively. The prevalence of postpartum depression was 22% (95% CI 15–29%). Multigravida women and women in the first and third trimesters of pregnancy were more vulnerable than other pregnant women. The assessment of the associations between the COVID-19 pandemic and mental health problems revealed that the pooled relative risks of anxiety and depression in pregnant women were 1.65 (95% CI: 1.25–2.19) and 1.08 (95% CI: 0.80–1.46), respectively.

**Conclusions:** The prevalence rates of mental disorders among pregnant and postpartum women during the COVID-19 pandemic were high. Timely and tailored interventions should be applied to mitigate mental problems among this population of women, especially multigravida women and women in the first and third trimesters of pregnancy.

## Introduction

The coronavirus disease 2019 (COVID-19) pandemic has become an unprecedented global crisis. All of us are battling the most powerful threat since the 21 century. However, a cure or an adequate safety vaccine has not yet been found or developed. Thus far, there is no indication that the COVID-19 pandemic will end quickly. Thus, pregnant women have to give birth during the COVID-19 pandemic. The pregnancy and the postpartum periods involve several drastic changes at the social, biological, and psychological levels in future mothers. Previous studies have painted a particularly difficult transition for pregnant and postpartum women. A systematic review and meta-analysis that involved 102 studies with 221,974 antenatal and postnatal women from 34 countries found that the pooled prevalence of anxiety among these participants was 15.2% (Dennis et al., 2017). Another systematic review and meta-analysis including 101 studies discovered that the pooled prevalence of depression among women in the perinatal period was 11.9% (Woody et al., 2017). The prevalence of postpartum depression was evaluated at 12.0% in a systematic review and meta-analysis that encompassed 58 studies with 37,294 postnatal women (Shorey et al., 2018). A meta-analysis involving data contributed by 11,002 pregnant women found that 45.7% of these women had poor sleep quality (Sedov et al., 2018). In 2020, pregnant and postpartum women have had to face the COVID-19 pandemic, its accompanying quarantine measures, and disruptions in medical practices. Many studies have found that during disasters or events, the prevalence rates of mental disorders among prenatal and postnatal women are significantly higher than those among the general population (Lechat, 1979; Vesga-López et al., 2008; Harville et al., 2010). Meeting the mental health needs of pregnant and postpartum women during the COVID-19 pandemic is a growing concern and a serious issue because a large body of robust evidence suggests that prenatal and postnatal mental disorders induce severe adverse influences on mothers, fetuses, and children. Prenatal and postnatal mental disorders induce disturbances in the physical activity, nutrition, and sleep of pregnant and postpartum women; these disturbances subsequently affect the mood of pregnant and postpartum women and the development of fetuses and children (Coussons-Read, 2013). Prenatal and postnatal mental disorders are correlated with physical disorders, such as preeclampsia (Zhang et al., 2013; Asghari et al., 2016), gestational hypertension (Zhang et al., 2013), and gestational diabetes (Gilbert et al., 2019); preterm birth (Grigoriadis et al., 2013, 2018; Ding et al., 2014); miscarriage (Accortt et al., 2015; Qu et al., 2017); low infant birth weight (Grigoriadis et al., 2013, 2018; Ding et al., 2014); fetal growth restriction (Grote et al., 2010; Ciesielski et al., 2015); lower Apgar scores at birth (Wu et al., 2020a); and socioemotional (Madigan et al., 2018), behavioral (Van den Bergh et al., 2005) and cognitive problems (Glover, 2014; Stein et al., 2014; Tarabulsy et al., 2014; MacKinnon et al., 2018), as well as changes in the brain structures and functions of infants and children (Sandman et al., 2015; Lebel et al., 2016; Adamson et al., 2018). Timely interventions are helpful in mitigating mental disorders (Kessler et al., 2007; Xiang et al., 2020). Knowing the effect of the COVID-19 pandemic on the mental health of pregnant and postpartum women, exploring the specific vulnerable groups among this population of women, and applying tailored interventions on the basis of data are urgent. The aims of this systematic review and meta-analysis are to quantify the influence of the COVID-19 pandemic on the mental health of pregnant and postpartum women, and to explore the specific vulnerable groups among this population of women.

## Materials and Methods

A systematic review and meta-analysis was conducted in accordance with the Preferred Reporting Items for Systematic Reviews and Meta-analyses (PRISMA) (Moher et al., 2009) and Meta-Analysis of Observational Studies in Epidemiology (MOOSE) (Stroup et al., 2000) guidelines. The review protocol was registered at PROSPERO as CRD42020210035.

### Search Strategy

Two authors (HY and YD) independently identified relevant studies that were published from January 1, 2019 to September 19, 2020 by searching the PubMed, EMBASE, and Web of Science databases. The following combined terms were applied in the search: (“pregnant woman” OR “breastfeeding women” OR “postpartum”) AND (“COVID-19” OR “2019 novel coronavirus disease” OR “2019-nCoV disease” OR “SARS-CoV-2”) AND (“mental health” OR “anxiety” OR “depression” OR “insomnia” OR “Stress Disorders, Post-Traumatic”). In addition, the reference lists of the identified records were hand-searched to find additional relevant studies.

### Study Selection Criteria

Studies were included if they reported the prevalence rates of depression, anxiety, insomnia, post-traumatic stress disorder (PTSD), and/or other mental health disorders among pregnant and/or postpartum women during the COVID-19 pandemic. Studies were also included if they reported data from which prevalence rates could be calculated. Letters, case reports, or reviews were excluded.

### Data Extraction and Quality Assessment

Two authors (HY and YD) independently extracted the following data from the studies that were eligible for this systematic review and meta-analysis: the name of the first author; the type of study; the time and locations of the studies; response rates; participants and the total number of participants; mean age; mean or median gestational age; the percentage of participants ≥ 35 years old; the percentage of nulliparous pregnant women; the percentages of pregnant women in the first, second, and third trimesters; the percentage of participants who were married or living with their partners; the percentage of participants who had a University degree or above; the used scales and applied cut-offs; and the percentages or the numbers of participants who were evaluated to be positive for mental disorders.

Two authors (HY and YD) independently evaluated the risk of bias of the studies included in the systematic review and meta-analysis. A third team member performed verification. Discrepancies were discussed and resolved among the 3 researchers. A modified form of the Newcastle–Ottawa scale was applied for quality assessment (Pappa et al., 2020). The modified form of the Newcastle–Ottawa scale has 5 items: 1, the representativeness of the sample (the number of pregnant or postpartum women ≥ 65% of the total sample); 2, the sample size of each study > 500 pregnant or postpartum women; 3, response rate > 80%; 4, the study applied validate measurement scales with appropriate cut-offs; and 5, appropriate and adequate statistics. Each item was given a score of 1 if the criterion was met or a score of 0 if the criterion was not met. Total scores of the studies ≥ 3 points indicated a low risk of bias. The total scores of studies assessed <3 points were regarded as at a high risk of bias.

### Data Analysis

Data analyses were performed by using Stata software version 12.0 (Stata Corp LP, College Station, USA). For the anticipated clinical heterogeneity, the pooled prevalence rates of anxiety, depression, insomnia, and other mental disorders with 95% confidence interval (CI) were calculated by using a random effects model. A random effects model is considered more suitable for meta-analyses with substantial heterogeneity than fixed effects model. Given that the included studies reported prevalence rates of mental disorders of close to 1 or 0, the Freeman–Tukey double arcsine transformation was performed before data pooling. *I*^2^ (significance level of *I*^2^ > 50%) and *Q*-tests (significance level of *P* < 0.05) were applied to evaluate heterogeneity across studies. Sensitivity analysis was conducted to evaluate the effect of each included study on the prevalence rates of mental disorders among pregnant or postpartum women by omitting each study and calculating the pooled prevalence rates of the remaining studies. Subgroup analysis was also performed on the basis of the used scales, study locations, parity, trimester, educational level, employment status, and mental disorder severity. Considering that some included studies reported the prevalence rates of mental disorders among pregnant or postpartum women during the COVID-19 pandemic and before the COVID-19 pandemic in the same study locations, a random effects model was utilized to evaluate summary relative risks (RRs) (during the COVID-19 pandemic vs. before the COVID-19 pandemic). Chi-squared statistic and *I*^2^ were applied to evaluate the homogeneity of effects across studies.

## Results

### Literature Search

**Figure 1:**
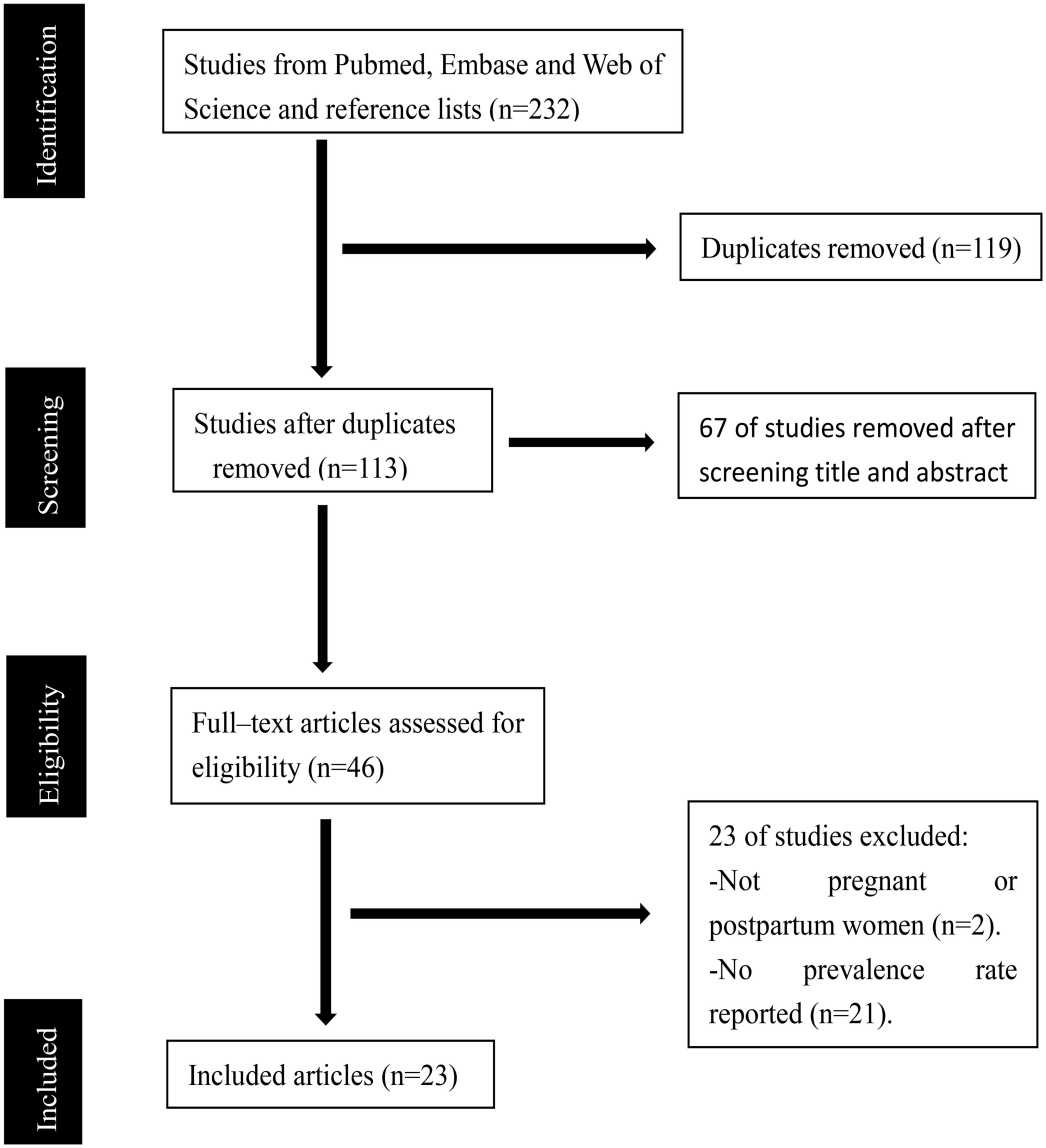
Preferred Reporting Items for Systematic Reviews and Meta-Analyses (PRISMA) study selection flow diagram.

Our initial search identified a total of 232 records (66 records in Pubmed, 104 records in Embase, and 62 records in Web of Science). A total of 119 articles were duplicates. After the duplicates were removed, 67 studies were excluded after reviewing their titles and abstracts. A total of 46 potentially relevant records were retrieved for detailed full-text evaluation. Finally, 23 articles met the selection criteria and were deemed to contain data relevant to the systematic review and meta-analysis. A PRISMA diagram detailing the process of article selection is shown in Figure 1.

### Characteristics of the Included Studies

A total of 23 studies (Ayaz et al., 2020; Berthelot et al., 2020; Ceulemans et al., 2020; Durankuş and Aksu, 2020; Farewell et al., 2020; Gu et al., 2020; He et al., 2020; Lebel et al., 2020; Li et al., 2020; Liu et al., 2020; Mappa et al., 2020; Matsushima and Horiguchi, 2020; Oskovi-Kaplan et al., 2020; Parra-Saavedra et al., 2020; Patabendige et al., 2020; Preis et al., 2020; Saccone et al., 2020; Sade et al., 2020; Silverman et al., 2020; Wu et al., 2020b; Yue et al., 2020; Zanardo et al., 2020; Zhang and Ma, 2020) performed with 20 569 participants (16,797 pregnant women and 3,772 postpartum women) during the COVID-19 pandemic and with 3,677 pregnant women before the COVID-19 pandemic were included in this systematic review and meta-analysis. A total of 19 studies (Ceulemans et al., 2020; Durankuş and Aksu, 2020; Farewell et al., 2020; He et al., 2020; Lebel et al., 2020; Li et al., 2020; Liu et al., 2020; Mappa et al., 2020; Matsushima and Horiguchi, 2020; Oskovi-Kaplan et al., 2020; Parra-Saavedra et al., 2020; Patabendige et al., 2020; Preis et al., 2020; Saccone et al., 2020; Sade et al., 2020; Silverman et al., 2020; Wu et al., 2020b; Yue et al., 2020; Zhang and Ma, 2020) were cross-sectional, and 4 (Ayaz et al., 2020; Berthelot et al., 2020; Gu et al., 2020; Zanardo et al., 2020) were case–control studies. Among the 23 studies, 7 were located in China (Gu et al., 2020; He et al., 2020; Li et al., 2020; Liu et al., 2020; Wu et al., 2020b; Yue et al., 2020; Zhang and Ma, 2020), 3 were implemented in America (Farewell et al., 2020; Preis et al., 2020; Silverman et al., 2020), 3 were performed in Turkey (Ayaz et al., 2020; Durankuş and Aksu, 2020; Oskovi-Kaplan et al., 2020), 3 were undertaken in Italy (Mappa et al., 2020; Saccone et al., 2020; Zanardo et al., 2020), 2 were conducted in Canada (Berthelot et al., 2020; Lebel et al., 2020), 1 took place in Belgium (Ceulemans et al., 2020), 1 occurred in Japan (Matsushima and Horiguchi, 2020), 1 was carried out in Colombia (Parra-Saavedra et al., 2020), 1 was accomplished in Sri Lanka (Patabendige et al., 2020), and 1 was done in Israel (Sade et al., 2020). The median questionnaire response rate was 88.05% (range 74.00%, 93.33%). The median percentage of the age of the participant ≥ 35 years old was 15.01% (range 10.94%, 44.44%). The median percentage of nulliparous pregnant women was 51.40% (range 34.50%, 71.55%). The median percentage of women who were married or living with their partners was 98.80% (range 90.00%, 100.00%). The median percentage of participants with a University degree or higher was 59.80% (range 10.00%, 93.00%). A summary of the characteristics of the 23 included studies is shown in Supplementary Table 1.

The scoring results obtained by using the modified form of the Newcastle–Ottawa scale are exhibited in Supplementary Table 2. Two studies were rated 2 points (Gu et al., 2020; Li et al., 2020), and 21 studies were rated ≥ 3 points.

### Anxiety Prevalence

**Figure 2:**
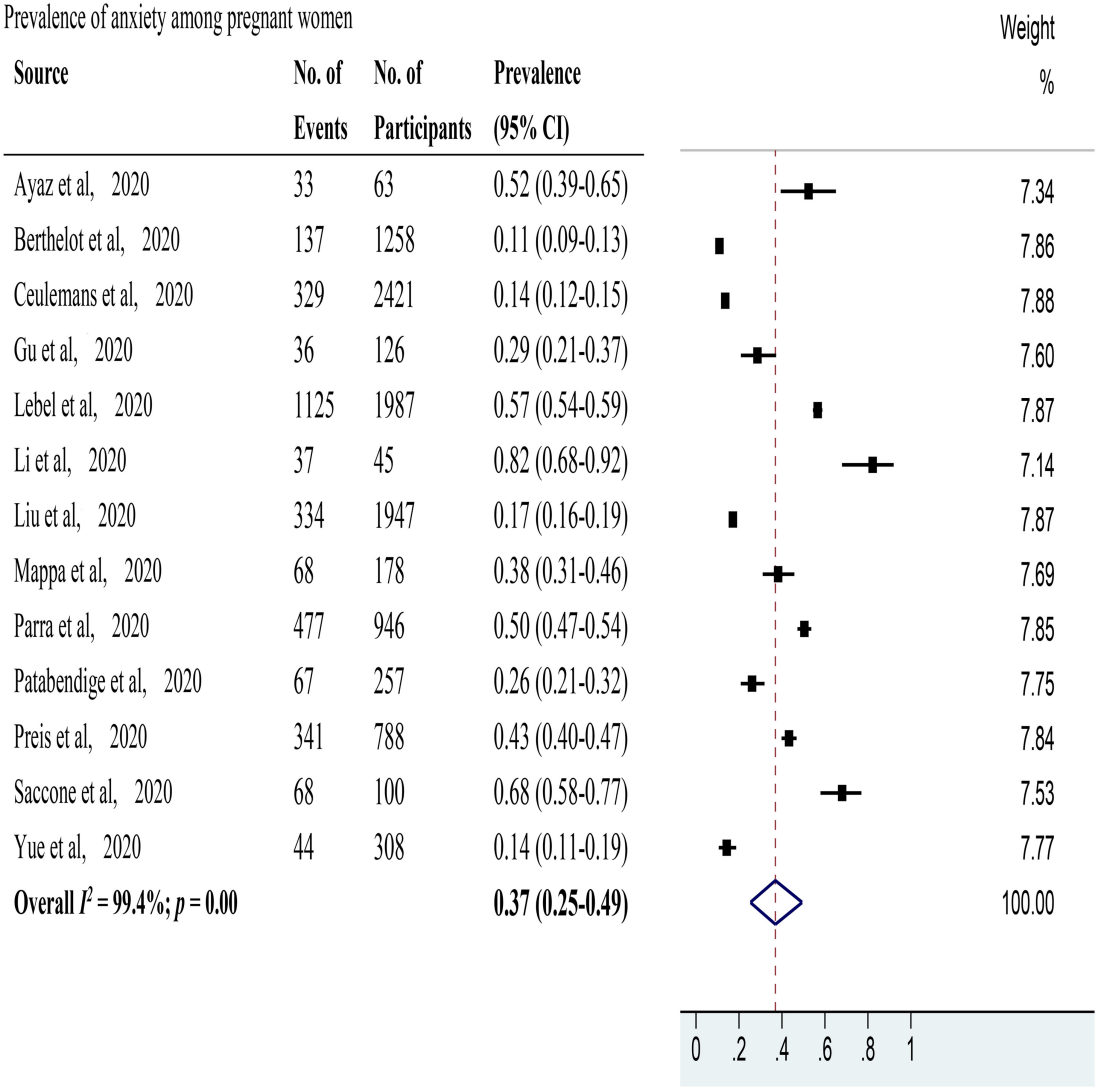
The pooled prevalence of anxiety among pregnant women during the COVID-19 pandemic.

Anxiety was evaluated in 13 studies (Ayaz et al., 2020; Berthelot et al., 2020; Ceulemans et al., 2020; Gu et al., 2020; Lebel et al., 2020; Li et al., 2020; Liu et al., 2020; Mappa et al., 2020; Parra-Saavedra et al., 2020; Patabendige et al., 2020; Preis et al., 2020; Saccone et al., 2020; Yue et al., 2020) with 10,424 pregnant women. The pooled prevalence of anxiety among pregnant women was 37% (95% CI 25–49%, *I*^2^ = 99.4%) as shown in Figure 2. After excluding studies with a high risk of bias, 11 studies with a low risk of bias (Ayaz et al., 2020; Berthelot et al., 2020; Ceulemans et al., 2020; Lebel et al., 2020; Liu et al., 2020; Mappa et al., 2020; Parra-Saavedra et al., 2020; Patabendige et al., 2020; Preis et al., 2020; Saccone et al., 2020; Yue et al., 2020) showed a pooled prevalence of anxiety among pregnant women of 34% (95% CI 22–47%, *I*^2^ = 99.4%). In sensitivity analysis, 5 studies (Berthelot et al., 2020; Ceulemans et al., 2020; Li et al., 2020; Saccone et al., 2020; Yue et al., 2020) affected the pooled prevalence of anxiety among pregnant women by over 2%. After excluding these 5 studies, the recalculated prevalence of anxiety among pregnant women was 39% (95% CI 25–53%, *I*^2^ = 99.1%). As for study locations (Supplementary Figure 1), 4 studies (Gu et al., 2020; Li et al., 2020; Liu et al., 2020; Yue et al., 2020) that were performed in China reported a pooled prevalence rate of anxiety among pregnant women of 33% (95% CI 18–50%, *I*^2^ = 96.9%), 2 studies (Berthelot et al., 2020; Lebel et al., 2020) undertaken in Canada disclosed a pooled prevalence rate of 37% (95% CI 35–38%, *I*^2^ = 99.9%), and 2 studies (Mappa et al., 2020; Saccone et al., 2020) conducted in Italy provided a pooled prevalence rate of 49% (95% CI: 43–55%, *I*^2^ = 96.1%). Each of the 5 remaining studies was carried out in a different country. For the used scales (Supplementary Figure 2), 2 studies (Ceulemans et al., 2020; Preis et al., 2020) applied the Generalized Anxiety Disorder 7-item Scale with a pooled prevalence rate of anxiety among pregnant women of 45% (95% CI: 17–74%, *I*^2^ = 99.4%), 2 (Liu et al., 2020; Yue et al., 2020) utilized the Self-Rating Anxiety Scale with a pooled prevalence rate of anxiety among pregnant women of 17% (95% CI: 15–18%, *I*^2^ = 42.8%), and 2 studies (Mappa et al., 2020; Saccone et al., 2020) applied state-trait anxiety inventory with a pooled prevalence rate of anxiety among pregnant women of 49% (95% CI: 43–55%, *I*^2^ = 96.1%). Each of the 7 remaining studies utilized a different scale. Two studies (Ayaz et al., 2020; Berthelot et al., 2020) reported the percentages of positive anxiety among pregnant women in the same location during and before the COVID-19 pandemic (Supplementary Figure 3). The pooled RR was 1.65 (95% CI: 1.25–2.19, *I*^2^ = 0.0%). The pooled prevalence rate of anxiety among postpartum women was not evaluated due to the limited data available.

### Depression Prevalence

**Figure 3:**
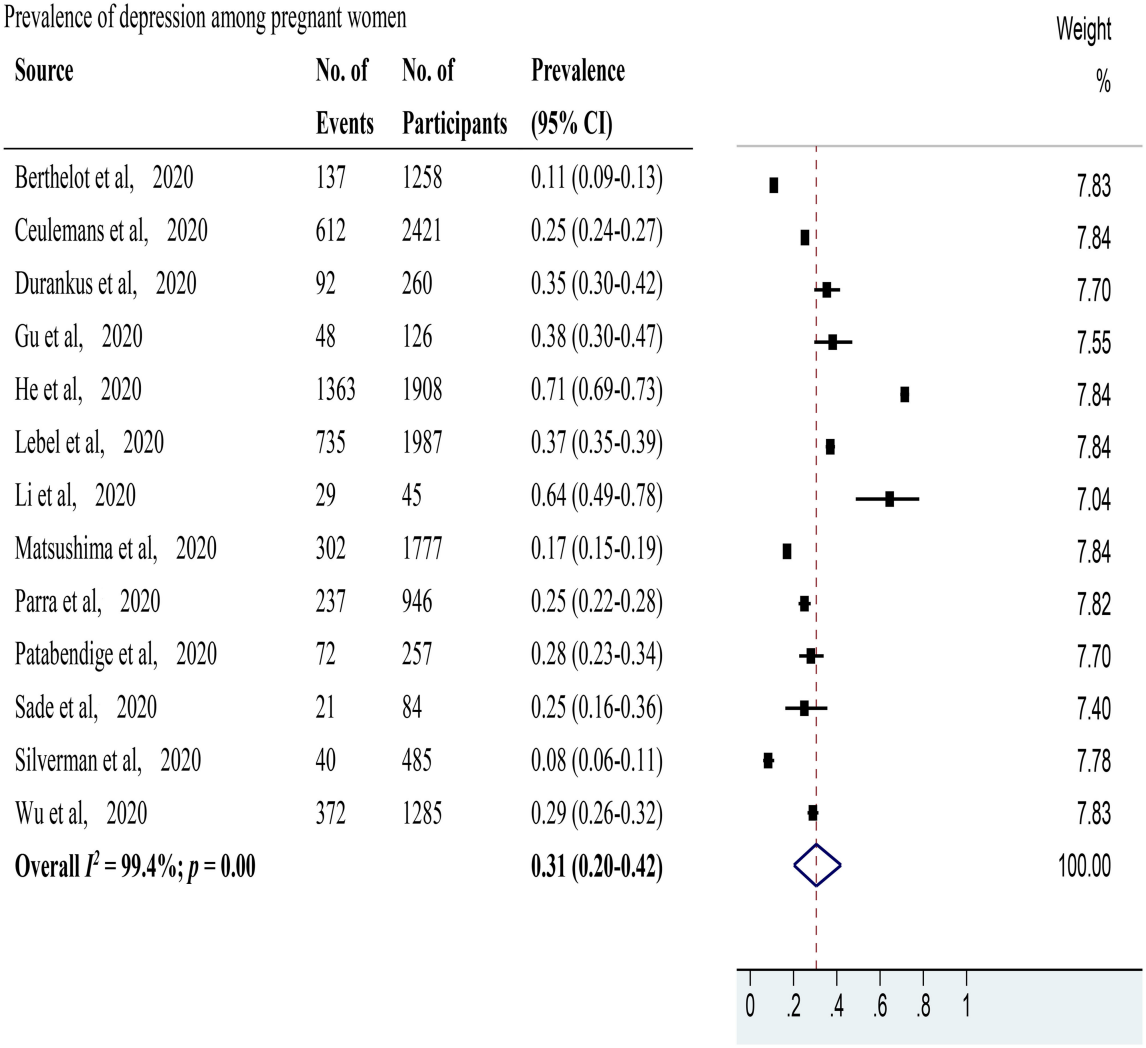
The pooled prevalence of depression among pregnant women during the COVID-19 pandemic.

**Figure 4:**
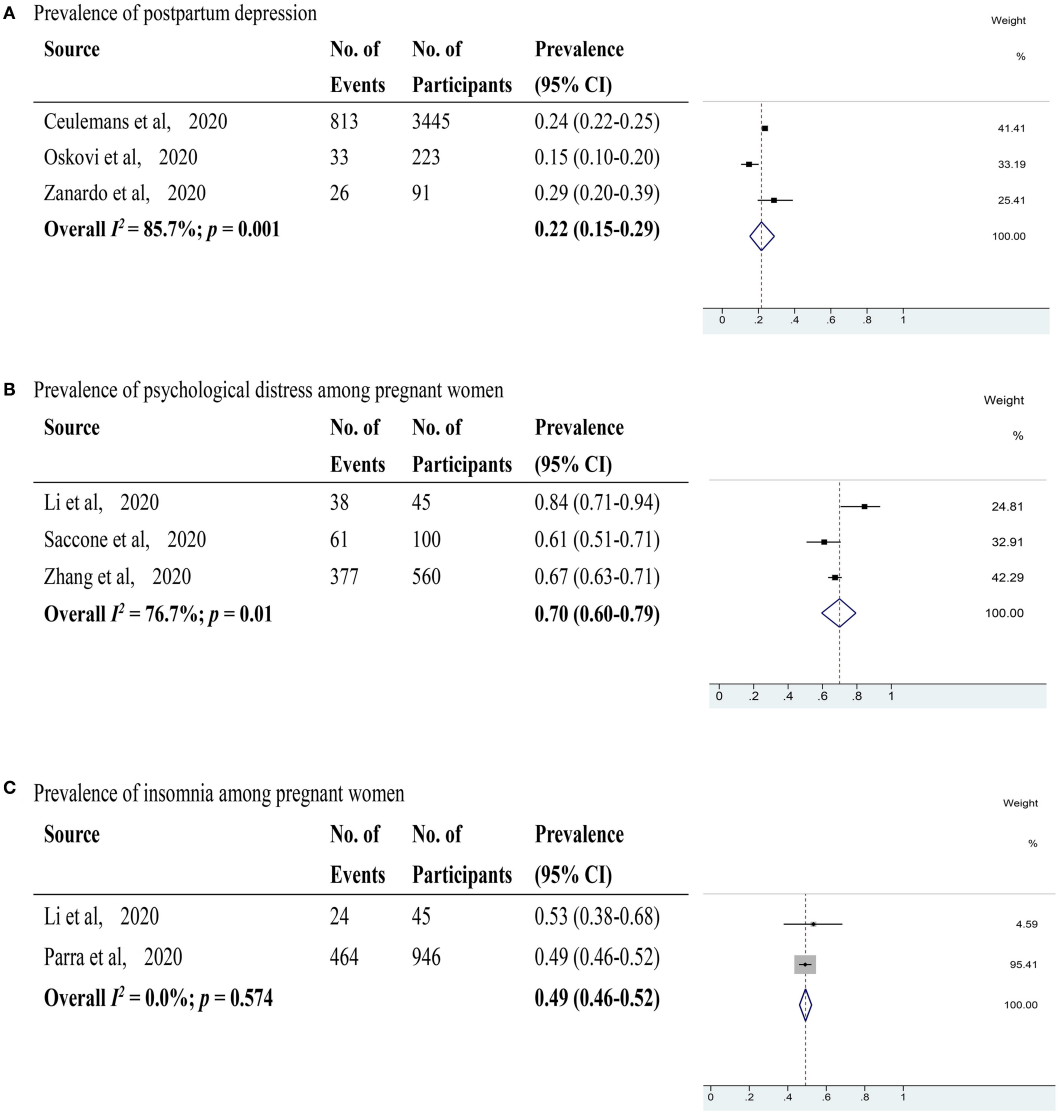
**(A)** The pooled prevalence of postpartum depression during the COVID-19 pandemic; **(B)** The pooled prevalence of psychological distress among pregnant women during the COVID-19 pandemic. **(C)** The pooled prevalence of insomnia among pregnant women during the COVID-19 pandemic.

Depression was evaluated in 13 studies (Berthelot et al., 2020; Ceulemans et al., 2020; Durankuş and Aksu, 2020; Gu et al., 2020; He et al., 2020; Lebel et al., 2020; Li et al., 2020; Matsushima and Horiguchi, 2020; Parra-Saavedra et al., 2020; Patabendige et al., 2020; Sade et al., 2020; Silverman et al., 2020; Wu et al., 2020b) with 12,839 pregnant women. The pooled prevalence of depression among pregnant women was 31% (95% CI 20–42%, *I*^2^ = 99.4%) as shown in Figure 3. After excluding studies with a high risk of bias, 11 studies with a low risk of bias (Berthelot et al., 2020; Ceulemans et al., 2020; Durankuş and Aksu, 2020; He et al., 2020; Lebel et al., 2020; Matsushima and Horiguchi, 2020; Parra-Saavedra et al., 2020; Patabendige et al., 2020; Sade et al., 2020; Silverman et al., 2020; Wu et al., 2020b) were included. These studies showed a pooled prevalence of depression among pregnant women of 27% (95% CI 17–40%, *I*^2^ = 99.5%). Through sensitivity analysis, 2 studies (He et al., 2020; Silverman et al., 2020) were found to affect the pooled prevalence of depression among pregnant women by over 2%. After excluding these 2 studies, the recalculated prevalence of depression among pregnant women was 29% (95% CI 23–35%, *I*^2^ = 97.8%). Regarding study locations (Supplementary Figure 4), 4 studies (Gu et al., 2020; He et al., 2020; Li et al., 2020; Wu et al., 2020b) were performed in China with a pooled prevalence rate of depression among pregnant women of 51% (95% CI 23–78%, *I*^2^ = 99.5%), and 2 studies (Berthelot et al., 2020; Lebel et al., 2020) were conducted in Canada with a pooled prevalence rate of 26% (95% CI 24–27%, *I*^2^ = 99.7%). Each of the 7 remaining studies took place in a different country. For used scales (Supplementary Figure 5), 7 studies (Durankuş and Aksu, 2020; He et al., 2020; Lebel et al., 2020; Matsushima and Horiguchi, 2020; Sade et al., 2020; Silverman et al., 2020; Wu et al., 2020b) applied the Edinburgh Postpartum Depression Scale with a pooled prevalence rate of depression among pregnant women of 31% (95% CI: 15–49%, *I*^2^ = 99.6%). Each of the 6 remaining studies utilized a different scale. Two studies (Sade et al., 2020; Wu et al., 2020b) reported the percentages of positive depression among pregnant women in the same location during and before the COVID-19 pandemic (Supplementary Figure 3). The pooled RR was 1.08 (95% CI: 0.80–1.46, *I*^2^ = 56.8%). Depression in postpartum women was evaluated in 3 studies (Ceulemans et al., 2020; Oskovi-Kaplan et al., 2020; Zanardo et al., 2020) with 3,759 postpartum women (Figure 4). The pooled prevalence of postpartum depression was 22% (95% CI 15–29%, *I*^2^ = 85.7%). Two studies (Oskovi-Kaplan et al., 2020; Zanardo et al., 2020) that assessed the prevalence of depression among postpartum women within 48 h after birth reported the pooled prevalence rate of 18% (95% CI 14–23%, *I*^2^ = 85.2%).

### Psychological Distress Prevalence

Psychological distress was evaluated in 3 studies (Li et al., 2020; Saccone et al., 2020; Zhang and Ma, 2020) with 705 pregnant women (Figure 4). The pooled prevalence rate of psychological distress among pregnant women was 70% (95% CI 60–79%, *I*^2^ = 76.7%). After excluding a study with a high risk of bias, 2 studies with a low risk of bias (Saccone et al., 2020; Zhang and Ma, 2020) showed a pooled prevalence rate of psychological distress among pregnant women of 66% (95% CI 63–70%, *I*^2^ = 30.6%). The pooled prevalence rate of psychological distress among postpartum women was not evaluated due to the limited data available.

### Insomnia Prevalence

Insomnia was evaluated in 2 studies (Li et al., 2020; Parra-Saavedra et al., 2020) with 991 pregnant women (Figure 4). The pooled prevalence rate of insomnia among pregnant women was 49% (95% CI 46–52%, *I*^2^ = 0.0%). However, 1 of the 2 studies used to calculate the prevalence rate of insomnia was assessed to have a high risk of bias.

### Subgroup Analysis

The subgroup analysis of the prevalence rate of anxiety among pregnant women was performed in accordance with the following categories: parity, trimester, educational level, employment status, and anxiety severity (Table 1 and Supplementary Table 3). Subgroup analysis for postpartum women was not conducted due to the limited data available. Parity data were provided by 2 studies (Mappa et al., 2020; Patabendige et al., 2020). The pooled prevalence rate of anxiety among primigravida women was 30% (95% CI 24–37%, *I*^2^ = 86.3%) and that among multigravida women was 31% (95% CI 26–37%, *I*^2^ = 46.3%). Trimester data were given by 2 studies (Patabendige et al., 2020; Saccone et al., 2020). The pooled prevalence rate of anxiety among pregnant women in the first trimester was 45% (95% CI 33–58%, *I*^2^ = 99.7%), that in the second trimester was 40% (95% CI 32–49%, *I*^2^ = 90.2%), and that in the third trimester was 35% (95% CI 27–43%, *I*^2^ = 95.0%). The data of educational level were available from 2 studies (Mappa et al., 2020; Patabendige et al., 2020). The pooled prevalence rate of anxiety among pregnant women with a University degree or above was 36% (95% CI 29–43%, *I*^2^ = 93.4%) and that with pregnant women with educational attainment below University education was 25% (95% CI 20–31%, *I*^2^ = 0.0%). Employment data were available in 2 studies (Mappa et al., 2020; Patabendige et al., 2020). The pooled prevalence rate of anxiety among employed pregnant women was 32% (95% CI 26–38%, *I*^2^ = 89.3%) and that among unemployed pregnant women was 23% (95% CI 18–29%, *I*^2^ = 70.4%). The data on mild anxiety in pregnant women were given in 5 studies (Ayaz et al., 2020; Ceulemans et al., 2020; Gu et al., 2020; Liu et al., 2020; Yue et al., 2020), and the pooled prevalence rate of mild anxiety among pregnant women was 24% (95% CI 11–40%, *I*^2^ = 99.0%). The data of moderate anxiety among pregnant women was available in 6 studies (Ayaz et al., 2020; Ceulemans et al., 2020; Gu et al., 2020; Lebel et al., 2020; Preis et al., 2020; Yue et al., 2020), and the pooled prevalence rate of moderate anxiety among pregnant women was 17% (95% CI 4–36%, *I*^2^ = 99.6%). The data of severe anxiety among pregnant women were provided in 6 studies (Ayaz et al., 2020; Ceulemans et al., 2020; Gu et al., 2020; Lebel et al., 2020; Preis et al., 2020; Yue et al., 2020), and the pooled prevalence rate of severe anxiety among pregnant women was 7% (95% CI 3–13%, *I*^2^ = 97.9%).

**Table 1:** Subgroup analysis of prevalence of anxiety and depression among pregnant women.

		**Anxiety**	**Depression**
Parity	Primigravida	**30%** 95% CI 24–37% *I*^2^ = 86.3%	**29%** 95% CI 24–35% *I*^2^ = 35.6%
	Multigravida	**31%** 95% CI 26–37% *I*^2^ = 46.3%	**34%** 95% CI 29–41% *I*^2^ = 79.4%
Trimester	First trimester	**45%** 95% CI 33–58% *I*^2^ = 99.7%	**21%** 95% CI 17–27% *I*^2^ = 51.2%
	Second trimester	**40%** 95% CI 32–49% *I*^2^ = 90.2%	**20%** 95% CI 17–22% *I*^2^ = 91.0%
	Third trimester	**35%** 95% CI 27–43% *I*^2^ = 95.0%	**22%** 95% CI 12–33% *I*^2^ = 96.6%

*95% CI, 95% confidence interval. The bold values are the prevalence rates of anxiety and depression among pregnant women according to different categories*.

The subgroup analysis of depression prevalence rates among pregnant women was conducted in accordance with parity and trimester due to the limited data available (Table 1). The parity data were available in 2 studies (Durankuş and Aksu, 2020; Patabendige et al., 2020). The pooled prevalence rate of depression among primigravida women was 29% (95% CI 24–35%, *I*^2^ = 35.6%) and that in multigravida women was 34% (95% CI 29–41%, *I*^2^ = 79.4%). The data for the first and second trimesters were provided in 2 studies (Matsushima and Horiguchi, 2020; Patabendige et al., 2020). The pooled prevalence rate of depression among pregnant women in the first trimester was 21% (95% CI 17–27%, *I*^2^ = 51.2%) and that among women in the second trimester was 20% (95% CI 17–22%, *I*^2^ = 91.0%). The data of pregnant women in the third trimester were given in 3 studies (Matsushima and Horiguchi, 2020; Patabendige et al., 2020; Wu et al., 2020b), and the pooled prevalence of depression in the third trimester was 22% (95% CI 12–33%, *I*^2^ = 96.6%).

The subgroup analysis of psychological distress and insomnia was not conducted due to the limited data available.

## Discussion

To the best of our knowledge, this is the first systematic review and meta-analysis to estimate the effect of the COVID-19 pandemic on the mental health of pregnant and postpartum women. A total of 23 studies conducted with 20,569 participants (16,797 pregnant women and 3,772 postpartum women) during the COVID-19 pandemic and with 3,677 pregnant women before the COVID-19 pandemic were included in this systematic review and meta-analysis. According to our analysis, the prevalence rates of anxiety, depression, psychological distress, and insomnia among pregnant women during the COVID-19 pandemic were 37% (95% CI 25–49%), 31% (95% CI 20–42%), 70% (95% CI 60–79%), and 49% (95% CI 46–52%), respectively. The prevalence of postpartum depression during the COVID-19 pandemic was 22% (95% CI 15–29%). The pooled RRs of anxiety and depression in pregnant women were 1.65 (95% CI: 1.25–2.19) and 1.08 (95% CI: 0.80–1.46), respectively, relative to those in pregnant women in the same locations during and before the COVID-19 pandemic. Through subgroup analysis, we found that multigravida women had higher prevalence rates of anxiety and depression than primigravida women during the COVID-19 pandemic. We also found that the prevalence of anxiety in pregnant women during the COVID-19 pandemic decreased throughout pregnancy, whereas the prevalence of depression followed a U pattern and was high in the first and third trimesters and lowest in the second trimester.

The pregnancy and postpartum periods involve several changes at the social, biological, and psychological levels in future mothers. Previous studies have found that pregnant and postpartum women have high prevalence rates of anxiety, depression, and insomnia (Dennis et al., 2017; Woody et al., 2017; Sedov et al., 2018; Shorey et al., 2018). During disasters or events, the prevalence rates of mental disorders in prenatal and postnatal women are significantly higher than those in the general population (Lechat, 1979; Vesga-López et al., 2008; Harville et al., 2010). In 2020, pregnant and postpartum women have to face the COVID-19 pandemic and its accompanying quarantine measures and disruptions in medical practices. Thus, adverse mental outcomes are amplified during the COVID-19 pandemic.

Before the COVID-19 pandemic, the estimated prevalence of anxiety among antenatal and postnatal women was 15.2% (Dennis et al., 2017), the pooled prevalence of depression among women in the perinatal period was 11.9% (Woody et al., 2017), the prevalence of postpartum depression was 12.0% (Shorey et al., 2018), and the prevalence of poor sleep quality was 45.7% among pregnant women (Sedov et al., 2018). In this systematic review and meta-analysis, we found that the prevalence rates of anxiety, depression, and insomnia among pregnant and postpartum women during the COVID-19 pandemic were higher than those before the COVID-19 pandemic. Pregnant and postpartum women also showed obvious higher prevalence rates of mental disorders during the COVID-19 pandemic than the general population. A systematic review and meta-analysis that included 50 studies found that the prevalence rates of anxiety, depression, psychological distress, and poor sleep quality among the general population were 26, 24, 26, and 34%, respectively (Krishnamoorthy et al., 2020). In this meta-analysis, we found that the pooled RRs of anxiety and depression in pregnant women were 1.65 (95% CI: 1.25–2.19) and 1.08 (95% CI: 0.80–1.46), respectively. These results verified that the COVID-19 pandemic induced increments in the prevalence rates of anxiety and depression.

Through subgroup analysis, we found that multigravida women had higher prevalence rates of anxiety and depression than primigravida women during the COVID-19 pandemic. Some previous studies which performed before the COVID-19 pandemic also reported similar results (Dipietro et al., 2008; Figueiredo and Conde, 2011). Multigravida women have to face several challenges, such as having an additional child, the reorganization of the existing parental system, and an increase in parental and financial responsibilities. These challenges may have a negative effect on the mental health of multigravida women. We also found that the prevalence of anxiety among pregnant women during the COVID-19 pandemic decreased throughout pregnancy (Woods-Giscombé et al., 2010; Figueiredo and Conde, 2011), whereas the prevalence of depression followed a U pattern (Lee et al., 2007; Bunevicius et al., 2009). Specifically, the prevalence of depression was high in the first and third trimesters and was the lowest in the second trimester. The increased prevalence rate of depression in the third trimester might be correlated with the proximity of giving birth. Moreover, these results might be induced by hormonal changes. Through the subgroup analysis of anxiety, we also found several results that contradicted the results of some previous studies and highlighted a higher prevalence of anxiety among pregnant women with a University degree or above than among pregnant women with low educational levels (Albrecht and Rankin, 1989; Qiao et al., 2009; Kannenberg et al., 2016) and a higher prevalence of anxiety among employed pregnant women than among unemployed pregnant women (Rubertsson et al., 2014). High educational level indicates high knowledgeability, which may amplify adverse effects on mental health during the COVID-19 pandemic. Employed pregnant women may face difficult situations, such the loss of jobs and earnings due to the COVID-19 pandemic. These difficult situations have a negative influence on mental health. We also found that the majority of pregnant women experienced mild and moderate anxiety, whereas severe anxiety was not common. Thus, timely and tailored interventions should be applied.

Some included studies also reported a high prevalence of fear (67.46%) (Gu et al., 2020), loneliness (60%) (Farewell et al., 2020), and PTSD (15.04%) (He et al., 2020) among pregnant women and a high RR of thoughts of self-harm among pregnant women in the same locations (during the COVID-19 pandemic vs. before the COVID-19 pandemic), (RR = 2.85; 95% CI: 1.70–8.85) (Wu et al., 2020b), although these data were not used in the final meta-analysis.

Meeting the mental health needs of pregnant and postpartum women during the COVID-19 pandemic is a serious issue. Numerous pieces of evidence suggest that prenatal and postnatal mental disorders exert heavy and lasting adverse influences on mothers, fetuses, and children. The induced adverse outcomes include preeclampsia (Zhang et al., 2013; Asghari et al., 2016), gestational hypertension (Zhang et al., 2013), and gestational diabetes of pregnant women (Gilbert et al., 2019); preterm birth (Grigoriadis et al., 2013, 2018; Ding et al., 2014); miscarriage (Accortt et al., 2015; Qu et al., 2017); low infant birth weight (Grigoriadis et al., 2013, 2018; Ding et al., 2014); fetal growth restriction (Grote et al., 2010; Ciesielski et al., 2015); lower Apgar scores at birth (Wu et al., 2020a); and socioemotional (Madigan et al., 2018), behavioral (Van den Bergh et al., 2005) and cognitive problems (Glover, 2014; Stein et al., 2014; Tarabulsy et al., 2014; MacKinnon et al., 2018), as well as changes in the brain structures and functions of infants and children (Sandman et al., 2015; Lebel et al., 2016; Adamson et al., 2018). This systematic review and meta-analysis highlighted the high prevalence rates of mental disorders among pregnant and postpartum women during the COVID-19 pandemic. The mental health of multigravida women and women in the first and third trimesters of pregnancy was vulnerable to the COVID-19 pandemic. Mental disorders in pregnant and postpartum women are the outcomes of a multivariate model with combined effects. This multivariate model comprises sociodemographic factors (age, parity, trimester, marital status, educational level, and socioeconomic status); stress (disaster or crisis, life events, marital satisfaction, and medical or obstetric complications); and support from partners, families, societies, and countries (Glazier et al., 2004; Farewell et al., 2020; Lebel et al., 2020; Mappa et al., 2020; Wu et al., 2020b; Yue et al., 2020). Although we found that the COVID-19 pandemic induced increments in the prevalence rates of mental disorders in pregnant and postpartum women, we cannot infer that the COVID-19 pandemic is the main factor across the factors influencing mental health of pregnant and postpartum women. Tailored interventions should be applied to mitigate mental problems in pregnant and postpartum women, especially multigravida women and women in the first and third trimesters of pregnancy.

This work is the first systematic review and meta-analysis that summarized existing literature on the mental health of pregnant and postpartum women, estimated the pooled prevalence rates of mental disorders, and highlighted vulnerable groups among the study population. Our review has certain limitations. One major drawback is the high heterogeneity across studies. The included studies applied different assessment tools and cut offs, although some studies used the same tools and cut offs. The studies' locations involved 10 countries, which face different severity levels of the COVID-19 pandemic. The included studies exhibit demographic differences such as the percentage of the age of the participants ≥ 35 years old, the percentage of nulliparous pregnant women, the percentage of women who were married or living with their partners, and the percentage of participants with a University degree or higher. Another limitation is that most of the included studies applied online questionnaires. This approach resulted in selection bias for the target population and lacked objectivity in the assessment outcomes. Moreover, the most of the included studies were cross-sectional. Thus, the long-term effects of the COVID-19 pandemic on the mental health of pregnant and postpartum women warrant additional longitudinal studies.

## Conclusion

This systematic review and meta-analysis summarized existing literature on the mental health of pregnant and postpartum women and highlighted the high prevalence rates of anxiety, depression, psychological distress, and insomnia among this population. Multigravida women and pregnant women in the first and third trimesters of pregnancy are highly vulnerable. Our findings are helpful for formulating tailored interventions to mitigate the effects of COVID-19 on the mental health of pregnant and postpartum women.

## Data Availability Statement

The original contributions presented in the study are included in the article/Supplementary Materials, further inquiries can be directed to the corresponding author/s.

## Author Contributions

HY designed the study and created the first draft of the manuscript. HY, YD, and WG performed the literature search, article selection, quality appraisal, and statistical analysis. YD and WG suggested improvements. All of the authors contributed to the final manuscript and submission.

## Conflict of Interest

The authors declare that the research was conducted in the absence of any commercial or financial relationships that could be construed as a potential conflict of interest.
