# Supplementary figures and images for: Mental Health of Pregnant and Postpartum Women During the Coronavirus Disease 2019 Pandemic: A Systematic Review and Meta-Analysis
Source: Front Psychol. 2020 Nov 25;11:617001. doi: 10.3389/fpsyg.2020.617001 (PMC7723850; doi:10.3389/fpsyg.2020.617001)

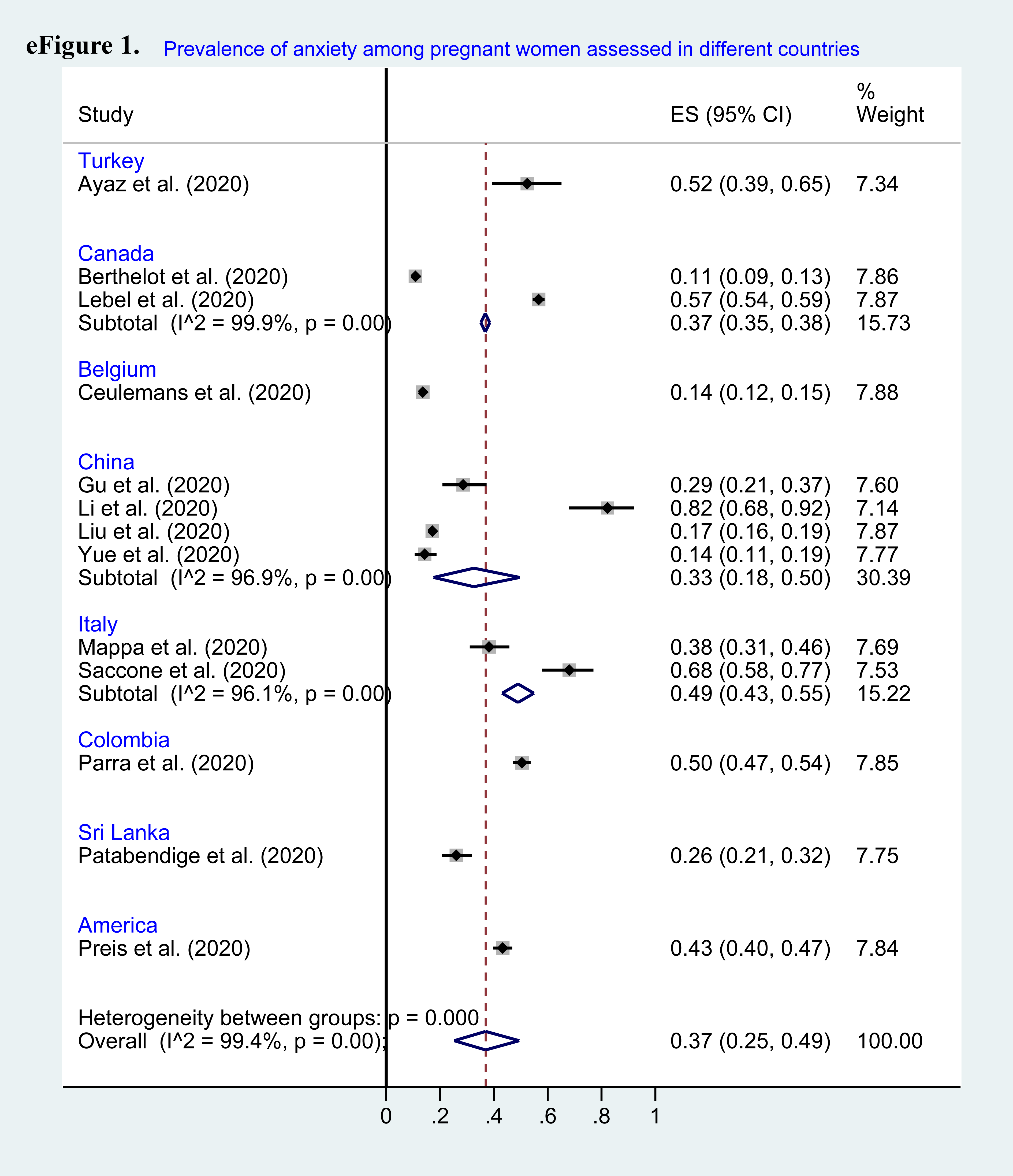

Supplement: Supplementary file 1 [file Image_1.TIF]

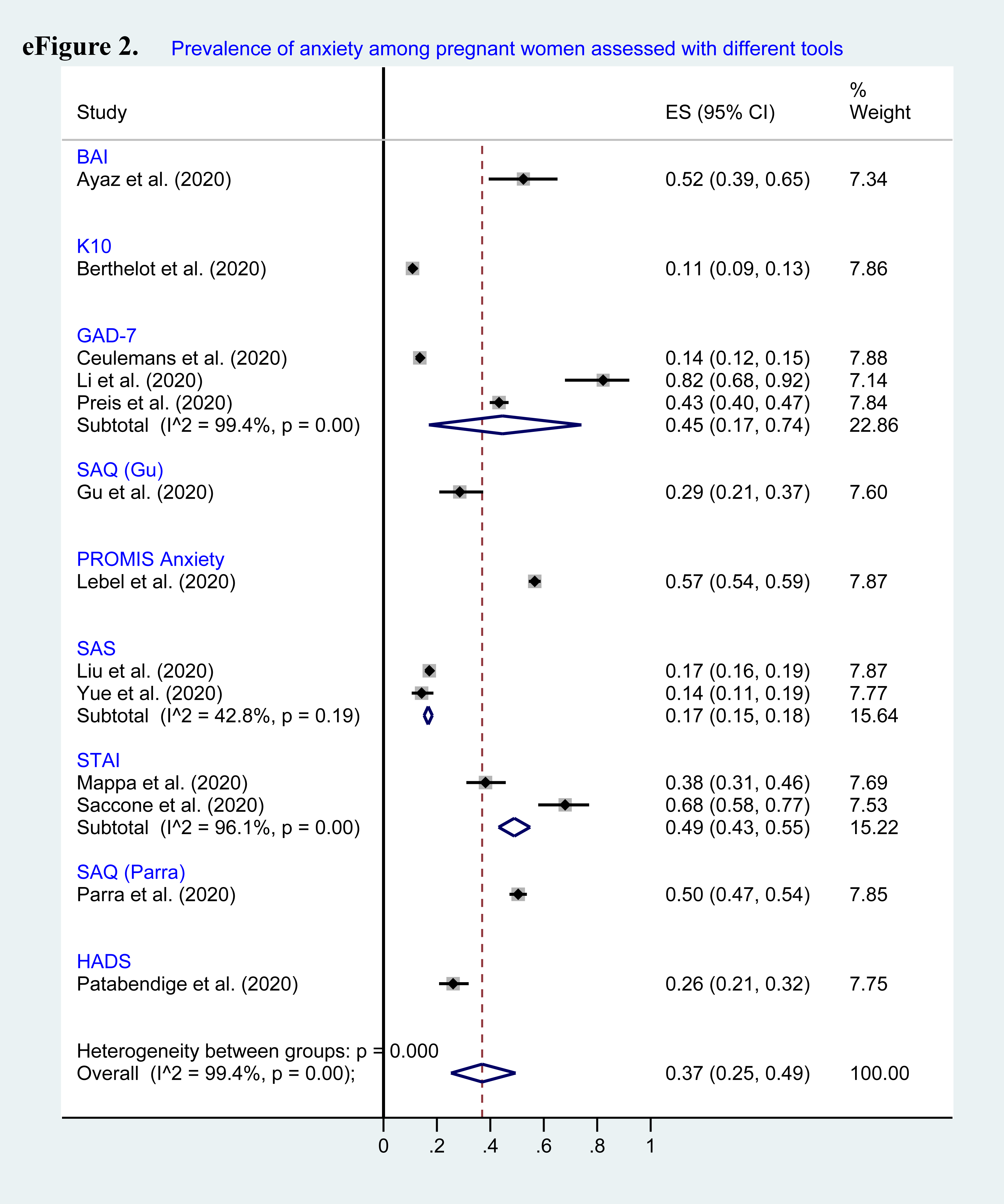

Supplement: Supplementary file 2 [file Image_2.TIF]

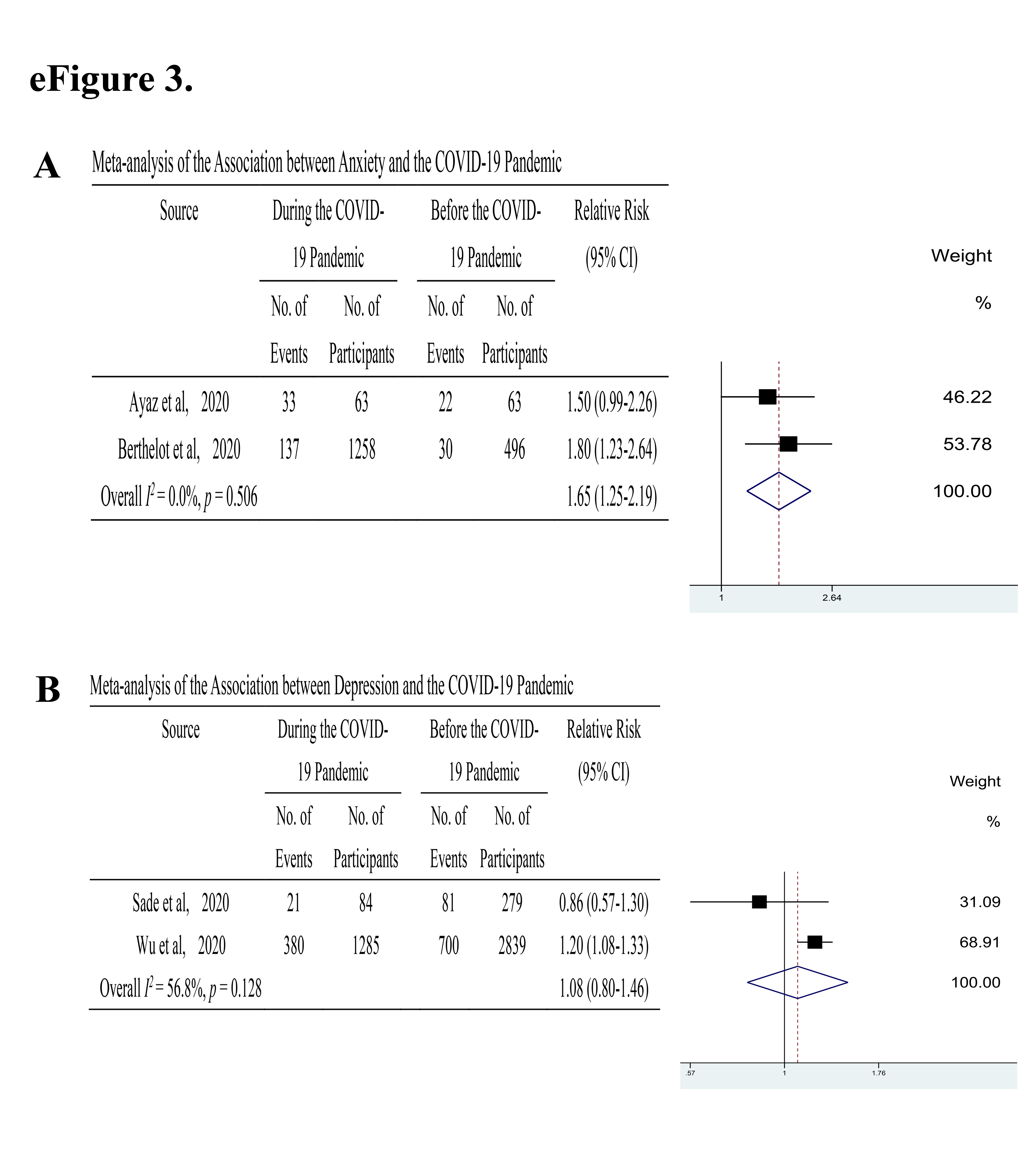

Supplement: Supplementary file 3 [file Image_3.TIF]

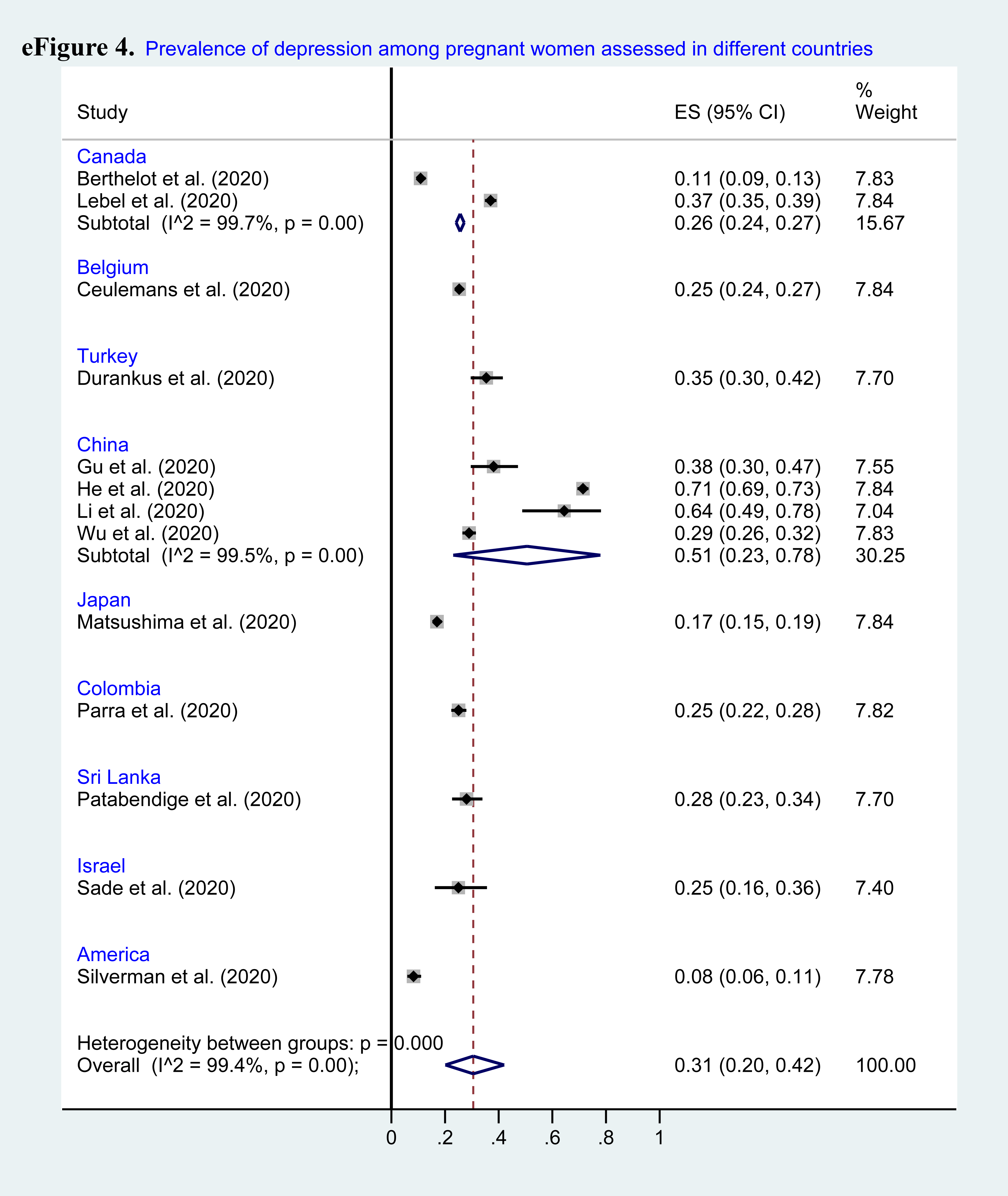

Supplement: Supplementary file 4 [file Image_4.TIF]

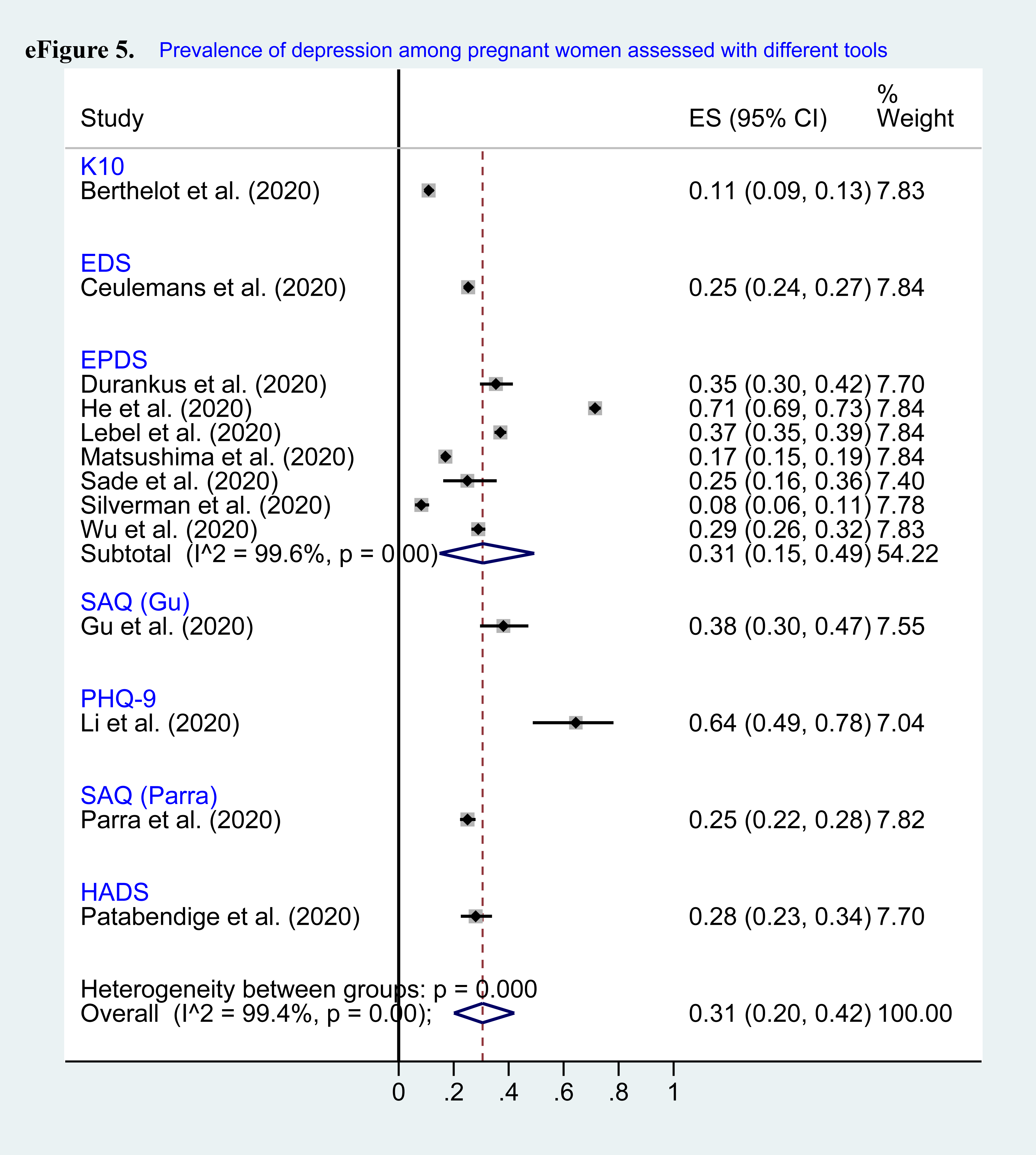

Supplement: Supplementary file 5 [file Image_5.TIF]
