# Supplementary material for: Mental Health of Pregnant and Postpartum Women During the Coronavirus Disease 2019 Pandemic: A Systematic Review and Meta-Analysis
Source: Front Psychol. 2020 Nov 25;11:617001. doi: 10.3389/fpsyg.2020.617001 (PMC7723850; doi:10.3389/fpsyg.2020.617001)
Supplement: Supplementary file 6 [file Table_1.DOCX]

eTable 1.

Characteristics of the included studies.

| Author | Participants | Study design | Dates (2020) | Location | Sample size | Response rate (%) | Mean age (years) | Mean or median gestational age (weeks) | Age ≥ 35(%) | Nulliparous (%) | Married or living with partner (%) | University degree or above (%) | Scales and cut–offs |
| --- | --- | --- | --- | --- | --- | --- | --- | --- | --- | --- | --- | --- | --- |
| Ayaz et al. | pregnant | case–control | NA | Turkey | 63 | NA | 30.4±5.3 | 32.5±7.0 | NA | NA | NA | NA | BAI |
| Berthelot et al. | pregnant | case–control | Apr 2–13 | Canada | 1258 | NA | 29.4±4.0 | 24.4±9.3 | NA | NA | NA | 93.20% | K10 ≥ 30 |
| Ceulemans et al. | pregnant, postpartum | cross–sectional | NA | Belgium | 5866 | NA | NA | NA | NA | NA | NA | NA | EDS ≥ 13, GAD–7 ≥ 10 |
| Durankus et al. | pregnant | cross–sectional | NA | Turkey | 260 | 81.80% | 29.6±3.8 | 7.0±5.9 | NA | 70.80% | NA | NA | EPDS ≥ 13 |
| Farewell et al. | pregnant, postpartum | cross–sectional | Mar–Apr | America | 27 | 87.10% | NA | NA | 44.44% | 44.44% | NA | NA | PHQ–2 ≥ 3, GAD–7 ≥ 10 |
| Gu et al. | pregnant | case–control | Jan–Feb | China | 126 | NA | NA | NA | NA | NA | NA | NA | SAQ |
| He et al. | pregnant | cross–sectional | Feb 13–16 | China | 1908 | NA | NA | NA | NA | NA | NA | NA | EPDS |
| Lebel et al. | pregnant | cross–sectional | Apr 5–20 | Canada | 1987 | NA | 32.4±4.2 | 22.5±8.4 | NA | 49.00% | 97.30% | NA | EPDS ≥ 13, PROMIS Anxiety ≥ 60 |

Continued.

Characteristics of the included studies.

| Author | Participants | Study design | Dates (2020) | Location | Sample size | Response rate (%) | Mean age (years) | Mean or median gestational age (weeks) | Age ≥ 35(%) | Nulliparous (%) | Married or living with partner (%) | University degree or above (%) | Scales and cut–offs |
| --- | --- | --- | --- | --- | --- | --- | --- | --- | --- | --- | --- | --- | --- |
| Li et al. | pregnant | cross–sectional | 25 Apr–9 May | China | 45 | NA | NA | NA | NA | NA | NA | NA | PHQ–9 ≥ 5, GAD–7 ≥ 5, ISI–7 ≥ 8, IES ≥ 8. |
| Liu et al. | pregnant | cross–sectional | Feb 3–9 | China | 1947 | NA | NA | NA | 10.94% | 71.55% | NA | 85.21% | SAS ≥ 50 |
| Mappa et al. | preganat | cross–sectional | March 9–10 | Italy | 178 | 89.00% | NA | 18.0 | NA | 56.80% | 98.80% | 39.90% | STAI ≥ 40 |
| Matsushima et al. | pregnant | cross–sectional | 31 May–6 Jun | Japan | 1777 | 74.00% | NA | NA | 28.25% | 65.17% | 96.40% | 80.70% | EPDS ≥ 13 |
| Oskovi et al. | postpartum | cross–sectional | Jun–20 | Turkey | 223 | NA | NA | NA | NA | NA | NA | 10.31% | EPDS ≥ 13 |
| Parra et al. | pregnant | cross–sectional | 13 Apr–May 18 | Colombia | 946 | 92.60% | NA | 24.0 | 15.01% | 44.00% | NA | 17.70% | SAQ |
| Patabendige et al. | pregnant | cross–sectional | 27 Apr–May 20 | Sri Lanka | 257 | NA | NA | NA | 14.70% | 35.00% | NA | 10.10% | HADS ≥ 8 |
| Preis et al. | pregnant | cross–sectional | the end of Apr | America | 788 | NA | 29.2±5.3 | 25.3±9.1 | NA | 45.90% | NA | NA | GAD–7 ≥ 10 |

Continued.

Characteristics of the included studies.

| Author | Participants | Study design | Dates (2020) | Location | Sample size | Response rate (%) | Mean age (years) | Mean or median gestational age (weeks) | Age ≥ 35(%) | Nulliparous (%) | Married or living with partner (%) | University degree or above (%) | Scales and cut–offs |
| --- | --- | --- | --- | --- | --- | --- | --- | --- | --- | --- | --- | --- | --- |
| Saccone et al. | pregnant | cross–sectional | 15 Mar–1 Apr | Italy | 100 | NA | NA | NA | NA | NA | NA | NA | IES ≥ 24,STAI > 36 |
| Sade et al. | pregnant | cross–sectional | 19 Mar–26 May | Israel | 84 | 93.33% | NA | 33.7 ± 5.1 | 17.90% | 34.50% | 90.40% | NA | EPDS ≥ 10 |
| Silverman et al. | pregnant | cross–sectional | 2 Feb–12 Jun | America | 485 | NA | NA | NA | NA | NA | NA | NA | EPDS ≥ 12 |
| Wu et al. | pregnant | cross–sectional | 21 Jan–9 Feb | China | 1285 | NA | 30.0 | NA | 14.60% | 68.80% | 98.90% | 79.70% | EPDS ≥ 10 |
| Yue et al. | pregnant | cross–sectional | Feb 16–21 | China | 308 | 81.10% | 31.0 ± 3.9 | 31.6 ± 2.2 | NA | NA | 100% | 93.20% | SAS > 50 |
| Zanardo et al. | postpartum | case–control | 8 Mar–3 May | Italy | 91 | NA | 33.7 ± 5.0 | 39.4 ± 1.1 | NA | 53.80% | 100% | 29.70% | EPDS > 12 |
| Zhang et al. | pregnant | cross–sectional | Feb–Mar | China | 560 | 93.30% | 25.8 ± 2.7 | NA | NA | NA | NA | NA | IES > 26 |

BAI = Beck Anxiety Inventory, K10 = 10-item Kessler Psychological Distress Scale, GAD-7 = Generalized Anxiety Disorder 7-item Scale, SAQ = self-administered questionnaire, PROMIS anxiety = PROMIS Anxiety Adult 7-item short form, SAS = Self-Rating Anxiety Scale, STAI = State-trait anxiety inventory, HADS = Hospital Anxiety and Depression Scale, 9, Edinburgh Depression Scale-EDS, EPDS = Edinburgh Postpartum Depression Scale, PHQ-2 = Patient Health Questionnaire–2, PHQ-9 = Patient Health Questionnaire-9, IES-7 = Impact of Event Scale-7, IES-R = Impact of Event Scale-Revised, IES = Impact of Event Scale, ISI-7 = Insomnia Severity Index-7.
