# Supplementary material for: Mental Health of Pregnant and Postpartum Women During the Coronavirus Disease 2019 Pandemic: A Systematic Review and Meta-Analysis
Source: Front Psychol. 2020 Nov 25;11:617001. doi: 10.3389/fpsyg.2020.617001 (PMC7723850; doi:10.3389/fpsyg.2020.617001)
Supplement: Supplementary file 7 [file Table_2.DOCX]

eTable 2.

Total score of each study assessed with modified Newcastle–Ottawa quality assessment scale

| Studies | Quality assessment scale | | | | | | Score | |
| --- | --- | --- | --- | --- | --- | --- | --- | --- |
|  | | 1 | 2 | 3 | 4 | 5 | |  |
| Ayaz et al. | | * | – | – | * | * | | 3 |
| Berthelot et al. | | * | * | – | * | – | | 3 |
| Ceulemans et al. | | * | * | – | * | * | | 4 |
| Durankus et al. | | * | – | * | * | * | | 4 |
| Farewell et al. | | * | – | * | * | * | | 4 |
| Gu et al. | | * | – | – | – | * | | 2 |
| He et al. | | * | * | – | – | * | | 3 |
| Lebel et al. | | * | * | – | * | * | | 4 |
| Li et al. | | – | – | – | * | * | | 2 |
| Liu et al. | | * | * | – | * | * | | 4 |
| Mappa et al. | | * | – | * | * | * | | 4 |
| Matsushima et al. | | * | * | – | * | * | | 4 |
| Oskovi et al. | | * | – | – | * | * | | 3 |
| Parra et al. | | * | * | * | – | * | | 4 |
| Patabendige et al. | | * | – | – | * | * | | 3 |
| Preis et al. | | * | * | – | * | * | | 4 |
| Saccone et al. | | * | – | – | * | * | | 3 |
| Sade et al. | | * | – | * | * | * | | 4 |
| Silverman et al. | | * | – | – | * | * | | 3 |
| Wu et al. | | * | * | – | * | * | | 4 |
| Yue et al. | | * | – | * | * | * | | 4 |
| Zanardo et al. | | * | – | – | * | * | | 3 |
| Zhang et al. | | * | * | * | * | * | | 5 |

1. Representativeness of sample (the number of pregnant or postpartum women ≥ 65% of total sample); 2. Sample size > 500 pregnant or postpartum women; 3. Response rate > 80%; 4. The study employed validate measurement tools with appropriate cut–offs; 5. Adequate statistics and no need for further calculation.
