# Supplementary material for: Mental Health of Pregnant and Postpartum Women During the Coronavirus Disease 2019 Pandemic: A Systematic Review and Meta-Analysis
Source: Front Psychol. 2020 Nov 25;11:617001. doi: 10.3389/fpsyg.2020.617001 (PMC7723850; doi:10.3389/fpsyg.2020.617001)
Supplement: Supplementary file 8 [file Table_3.DOCX]

eTable 3.

Subgroup analysis of prevalence of anxiety among pregnant women.

| Categories |  | Pooled prevalence (%) | 95% CI, I^2^ |
| --- | --- | --- | --- |
| Educational level | University degree or above | 36% | 29%–43%, I^2^ = 93.4% |
|  | Educational attainment below university education | 25% | 20%–31%, I^2^ = 0.0% |
| Employment status | employed | 32% | 26%–38%, I^2^ = 89.3% |
|  | unemployed | 23% | 18%–29%, I^2^ = 70.4% |
| Anxiety severity | mild | 24% | 11%–40%, I^2^ = 99.0% |
|  | moderate | 17% | 4%–36%, I^2^ = 99.6% |
|  | severe | 7% | 3%–13%, I^2^ = 97.9% |

95% CI = 95% confidence interval.
